# Inferring Predator Behavior from Attack Rates on Prey-Replicas That Differ in Conspicuousness

**DOI:** 10.1371/journal.pone.0048497

**Published:** 2012-10-31

**Authors:** Yoel E. Stuart, Nathan Dappen, Neil Losin

**Affiliations:** 1 Museum of Comparative Zoology and Department of Organismic and Evolutionary Biology, Harvard University, Cambridge, Massachusetts, United States of America; 2 Department of Biology, University of Miami, Coral Gables, Florida, United States of America; 3 Department of Ecology and Evolutionary Biology, University of California Los Angeles, Los Angeles, California, United States of America; University of Sussex, United Kingdom

## Abstract

Behavioral ecologists and evolutionary biologists have long studied how predators respond to prey items novel in color and pattern. Because a predatory response is influenced by both the predator’s ability to detect the prey and a post-detection behavioral response, variation among prey types in conspicuousness may confound inference about post-prey-detection predator behavior. That is, a relatively high attack rate on a given prey type may result primarily from enhanced conspicuousness and not predators’ direct preference for that prey. Few studies, however, account for such variation in conspicuousness. In a field experiment, we measured predation rates on clay replicas of two aposematic forms of the poison dart frog *Dendrobates pumilio*, one novel and one familiar, and two cryptic controls. To ask whether predators prefer or avoid a novel aposematic prey form independently of conspicuousness differences among replicas, we first modeled the visual system of a typical avian predator. Then, we used this model to estimate replica contrast against a leaf litter background to test whether variation in contrast alone could explain variation in predator attack rate. We found that absolute predation rates did not differ among color forms. Predation rates relative to conspicuousness did, however, deviate significantly from expectation, suggesting that predators do make post-detection decisions to avoid or attack a given prey type. The direction of this deviation from expectation, though, depended on assumptions we made about how avian predators discriminate objects from the visual background. Our results show that it is important to account for prey conspicuousness when investigating predator behavior and also that existing models of predator visual systems need to be refined.

## Introduction

The behaviors of predators, particularly their strategies for sampling unfamiliar prey, influence the evolution of prey defenses like aposematism, crypsis, and color polymorphism [1]. Accordingly, such predator behaviors have received considerable attention in the empirical and theoretical literature. Often, the empirical study of predators’ responses to novel prey involves the manufacture of artificial prey replicas that vary in color or pattern. These replicas are then exposed to predators in an experimental arena or in nature to infer the behavior of a specific predator species or an entire guild of predators [2], [3], [4], [5], [6].

Different replica color forms may vary in their conspicuousness to predators, however, confounding attempts to link variation in replica color pattern to variation in predator behavior [7], [8], [9]. For example, if a novel color form is attacked at a higher rate than an established form, it may be impossible to determine, without controlling for differences in conspicuousness, whether this difference in predation rate is driven by a predator’s behavioral preference for the novel color form or simply by a greater visual detection rate of the novel form. Because an understanding of predator behavior hinges on this interplay between prey detection and the subsequent predator response, it is crucial to account for differences in detectability among replicas. Such differences, however, are seldom considered (but see [10], [11], [12], [13]). In this study, we adjusted for differences in conspicuousness among prey replica types by explicitly incorporating a model of a predator’s visual system before inferring predator behavior.

We focused on the guild of avian predators at La Selva Biological Station (LSBS) in northeastern Costa Rica and used *Dendrobates pumilio*, the strawberry poison dart frog, as a model prey species. *Dendrobates pumilio* is a small, Neotropical frog whose conspicuous color pattern, toxic skin secretions, and diurnal activity suggest that it is aposematically colored [14], [15]. We used plasticine-clay replicas to simulate the introduction of an aposematic yellow-and-orange color form of *D. pumilio* – normally found in the Bocas Del Toro region of Panama – into the LSBS forest, where only a red-and-blue form is typically found. We compared predation rates on replicas of this novel aposematic form to rates on the familiar local aposematic form and two cryptic color forms.

To determine whether variation in predation rate among color forms can be explained merely by differences in replica visual contrast (i.e. predators attack indiscriminately upon detection) or, alternatively, whether predators are making behavioral decisions after detection to avoid or attack each form, we adjusted our null predictions for predation rates by incorporating data on the visual contrast to the avian eye of each replica color form against a leaf litter background [7], [16]. We found that our replicas do differ from one another in conspicuousness to the avian eye and that attack rates do not follow differences in conspicuousness among replicas, suggesting that predators are making post-detection decisions to avoid or attack prey.

**Figure 1:**
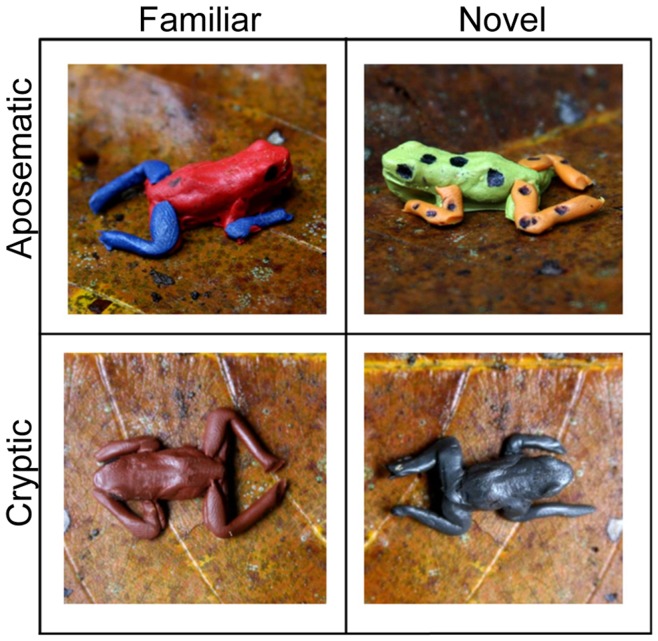
The four replicas. The familiar, aposematic (“red”) form resembles the *Dendrobates pumilio* form found at La Selva Biological Station (LSBS). The familiar, cryptic (“brown”) form resembles *Eleutherodactylus* spp. found at LSBS. The novel, aposematic (“yellow”) form resembles a form of *D. pumilio* from Bocas del Toro, Panama. The novel, cryptic (“black”) form resembles no frog found at LSBS.

## Materials and Methods

### Field Experiment

We conducted our experiment at La Selva Biological Station (LSBS) from May 17–29, 2008. LSBS encompasses 1500 hectares of primary and secondary lowland tropical wet forest (*sensu* [17]) on the Caribbean slope of Costa Rica. Using a mold formed from a preserved specimen of *D. pumilio*, we crafted frog replicas from precolored, non-toxic Sculpey® brand polymer clay (Polyform Products Company, IL, USA). Modeling clay is often used in predation experiments because predators that attack a replica leave identifiable impressions in the clay (e.g., [2], [11], [13]).

**Table 1:** Number of replicas deployed, recovered, and attacked.

replica color	replicas deployed	replicas recovered	total attacks	avian attacks	rodent attacks	un-attributed attacks
black	600	569	31	17	6	8
brown	600	577	26	10	5	11
red	600	597	18	12	1	5
yellow	600	592	33	19	7	7
sum	2400	2335	108	58	19	31

We manufactured 200 frog replicas that resembled the local *D. pumilio* form with a red body and blue limbs (i.e., an aposeme assumed to be familiar to LSBS predators) and 200 frog replicas that resembled a form of *D. pumilio* novel to LSBS but found on Isla Colon, Bocas Del Toro, Panama, with a yellow body, orange limbs and black spots [18] (Fig. 1). This aposeme is unlike any other frog at LSBS [19] and is assumed to be unfamiliar to predators at LSBS. In addition, we made 200 brown replicas colored to resemble one of several small *Eleutherodactylus spp.* leaf-litter frogs found at LSBS (i.e., a cryptic form familiar to predators at LSBS) and 200 black replicas (i.e., a cryptic form unfamiliar to predators at LSBS; [19]). We used a black permanent marker to place black eyespots on the replicas and to draw the black dorsal spots on the Isla Colon replicas. Hereafter, for brevity, we refer to each replica color form by its main body color (red, yellow, brown, and black, respectively). Saporito et al. [15] found that Sculpey® clay exhibits low ultraviolet (UV) reflectance. Because previous studies have shown that *D. pumilio* also has low reflectance in the UV range [20], we followed Saporito et al. [15] and mixed clay colors in proportions that best matched live frog colors according to the visual assessment of the authors (three males with normal color vision). We also compared spectrometric measurements of our red clay (the main color of the dorsum of the local aposematic form) against spectrometric measurements of the dorsum of live red-and-blue *D. pumilio* morphs taken by Summers et al. [20]. Reflectance values of live frogs remain near zero until a sharp peak between 625 and 675 nm (Fig. 12 in ref. [20]), matching reflectance spectra for the red clay, which remain relatively flat until a sharp peak around 625 nm (Fig. S1). Given the similarity in reflectance spectra for red clay and live frogs, we assume that predators at La Selva are likely to be “familiar” with the red replicas and will treat them like live, local frogs.

**Figure 2:**
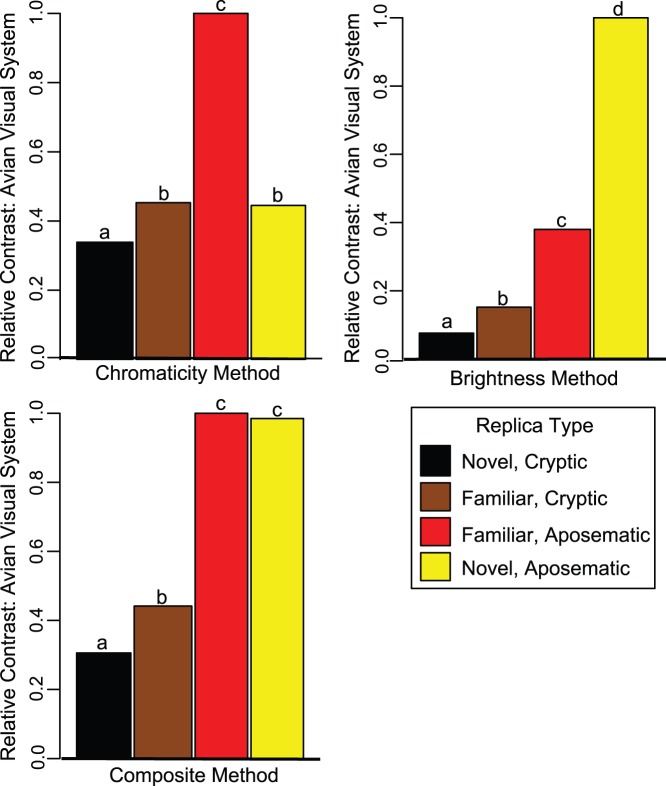
Relative contrast estimates for the three visual contrast methods under an avian visual model, scaled to 1. Estimates are not directly comparable among methods. Color forms assigned different letters differ significantly from each other after Bonferroni correction (Table S1).

**Figure 3:**
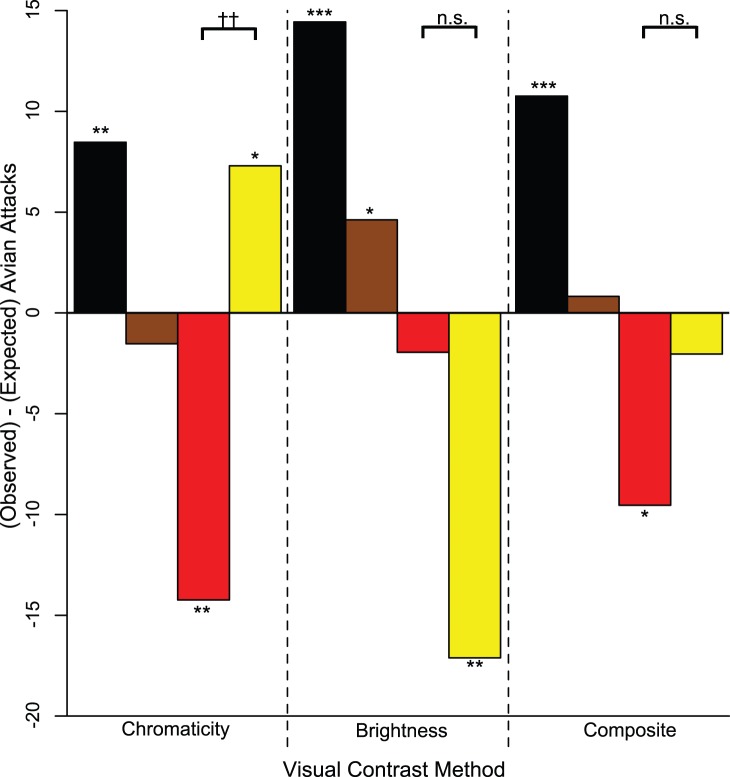
Observed-minus-expected avian attacks. Expected attack distributions were generated using the three visual contrast methods. Negative observed-minus-expected values signify that a color form was attacked less than expected and vice versa. Colors follow the legend in Fig. 2. Stars denote significant departures from the expected attack distribution. ‘*’: *P*<0.05, ‘**’: *P*<0.01. Crosses denote significant departures from the expected attack distribution from pairwise comparison of red vs. yellow replica attack rates. ††: *P*<0.01, n.s.: not significant.

We conducted six predation trials over 12 days with each trial in a unique location along LSBS’s dendritic trail system. Each trial consisted of ten 60 m transects; transects were spaced 50 m apart. Each transect contained 40 regularly spaced frog replicas placed directly onto the forest floor in a regular, alternating color order, haphazardly with respect to the background substrate. The density of replicas along study transects was realistic given the high density of *D. pumilio* in nature [21]. To aid our recovery searches, we ran clear fishing line approximately one meter above each transect and placed each frog replica approximately one meter to the right of the fishing line.

We collected the replicas after they had been in the forest for 48 hours. We inspected each recovered replica for evidence of predation and classified predation events as avian, rodent, or un-attributable. Avian predators left beak shaped piercings and impressions in the clay; rodent predators left easily identifiable incisor marks (Fig. S2).

During collection, we recorded how many seconds it took for a human researcher to locate each individual frog replica. These search time data were collected as a proxy for replica conspicuousness, at least to the human eye (see Text S1). If a replica was not found after 180 seconds, it was considered missing. We chose 180 s as our cutoff because a February 2008 pilot study determined that replicas not found by 180 s would likely not be found at all. Following recent studies, missing replicas were not included in subsequent analyses since their fates could not be determined reliably [15], [22].

### Avian Visual-model-based Estimates of Replica Conspicuousness

#### (i) Spectrometric measurements

To account for differences in visual conspicuousness among replica color forms, we took spectrometric measurements of the clay color mixtures used in the replicas as well as leaf litter samples collected from the replica transects. At each transect, a 25 cm×25 cm quadrat was thrown haphazardly onto the forest floor, and four representative leaves were selected from this quadrat. On each leaf we measured reflectance at three locations (averaging three spectrometer readings per location), and then calculated an average reflectance measurement for each leaf (Fig. S3).

To quantify clay colors, we measured five samples of each standardized color mixture (red, orange, yellow, blue, brown, and black) used in the replicas, and one pen mark from a black permanent marker on a yellow clay background. We averaged three spectrometer readings per clay sample and then averaged across the five samples to obtain one spectrum representing each standard color mixture. For all color measurements, we used an Ocean Optics USB-2000 spectrometer with an R-400 reflectance probe and PX-2 pulsed xenon light source, and Optics OOIBase 32 v2.0.6.5 software (Ocean Optics, Inc., FL, USA, 2002). We used white and dark standards to calibrate the spectrometer before measurements were taken.

#### (ii) Replica conspicuousness to the avian eye

We focus on the avian predator assemblage because birds were likely the most common visual predators of *D. pumilio* at LSBS [15], [23], [24]. The avian visual system has both double-cones, which seem to be used in brightness and motion detection, and four single-cone classes that are used for color discrimination [25]. In general, bird eyes can be divided into two types according to their single-cone classes [26]: V-type and U-type; the latter type has greater sensitivity to UV wavelengths [26]. We used a model of the UV-insensitive, V-type avian visual system for this study [26], [27], assuming that the ambient light profile of LSBS’s forest understory matched the UV-poor “forest shade” profile of other lowland tropical forest sites [28]. Results for the U-type avian visual system are qualitatively similar and not presented below. Moreover, our results do not change qualitatively if an alternative ambient light profile [28] is used in our models instead.

Animals may use chromatic information independent of brightness, brightness information independent of chromaticity, or both chromatic and brightness information to detect prey against the visual background [29]. Therefore, we calculated visual contrast between frog replicas and the leaf litter background in three ways by extracting from the cone excitation data: (1) only chromatic information, (2) only brightness information, and (3) both chromatic and brightness information. The computer code for extracting color information from the reflectance spectra is available upon request. Cone sensitivity functions [26] were derived from cone excitation values for ten avian species with a V-type eye (see Table 1 in ref. [26]).

1. *Chromaticity-only contrast*: We transformed the four avian single-cone excitation values for each reflectance spectrum into three-dimensional coordinates in a tetrahedral color space using a method developed by [26]. We then calculated color contrast as the Euclidean distance between clay color and individual leaf color in this color space.
2. *Brightness-only contrast*: To calculate brightness contrast, we calculated the difference in double-cone excitation between clay colors and individual leaf colors. Double-cone excitation values came from the reflectance spectra and a cone function derived from empirical measurements of double cone photoreceptor sensitivities and oil droplet absorbance spectra [30], [31], [32].
3. *Chromaticity+brightness contrast*: Because chromaticity and brightness contrasts were estimated using different cone types, we normalized contrasts under each method to one. Then, under the assumption that chromaticity contrast and brightness contrast contribute equally to object-background discrimination, we summed the chromaticity and brightness contrast proportions to obtain a composite contrast measure.

Hereafter, we refer to these three distance-based contrast measures as the “chromaticity,” “brightness,” and “composite” measures, respectively. To calculate an overall visual contrast score for each replica color and contrast method (chromaticity, brightness, and composite), we averaged the clay/leaf contrast scores across all leaf samples. Because aposematic replicas incorporated more than one clay color, we estimated whole-replica contrast under each contrast method by calculating the contrast between each individual clay color and the leaf litter background, and then taking a weighted average of these contrast values according to the area covered by each color on the replica (areas measured from digital photographs of frog replicas viewed dorsally).

#### (iii) Analysis

We used a Chi-square (χ^2^) test of independence to test for differences in absolute predation rates (i.e. regardless of contrast) among color forms. Then, to ask whether predation rates are proportional to the contrast of each form (i.e. do differences in attack rates match differences in conspicuousness?), we generated an expected attack distribution given replica contrast for each color form under each contrast method according to the equation:
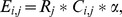
where *i* is the contrast method (chromaticity, brightness, or composite), *j* is the replica color (red, yellow, brown, or black), *E_i,j_* is the expected number of attacks, *R_j_* is the number of replicas recovered, *C_i,j_* is the visual contrast value, and *α* is a constant of proportionality that ensures that the total number of expected attacks is equal to the total number of observed attacks. Finally, we compared the expected attack distributions to the observed distribution using a χ^2^ test of independence.

## Results

### Predation Rates

We recovered 2335 of the 2400 replicas placed in the forest during our experiment: 569 black (novel, cryptic) replicas, 577 brown (familiar, cryptic) replicas, 597 red (familiar, aposematic) replicas, and 592 yellow (novel, aposematic) replicas (Table 1). Recovery times for frog replicas were quite variable within colors. Harmonic mean search time was 6.10 s for black replicas, 4.52 s for brown replicas, 2.20 s for yellow replicas, and 1.62 s for red replicas). Mean search time differed significantly between each pair of color forms (Fig. S4; Kruskal-Wallis Test: χ^2^_3_ = 735.66, *P*<0.001; post-hoc Behrens-Fisher test at α = 0.05: all pairwise contrasts *P*<0.001).

Of the recovered replicas, 108 (5%) were attacked; we attributed 58 attacks to birds, 19 attacks to rodents, and 31 attacks were of un-attributable origin (Table 1). Before correcting for contrast differences, replica color forms did not differ in overall predation rate (χ^2^_3_ = 5.61, *P* = 0.132, data pooled across all trials) or avian predation rate (χ^2^_3_ = 3.78, *P* = 0.286, data pooled across all trials). Similar results were found when each trial was considered individually (not shown).

Avian predators likely have home ranges that overlap multiple replicas of each type within a single transect and may overlap two or more transects as well. Therefore, it is possible that a single individual could be responsible for multiple attacks on adjacent individuals, violating statistical assumptions of independence. We performed our analyses again after removing any sets of replicas that were attacked consecutively [15]. The results remain qualitatively unchanged (not shown).

The data collected in this study, as well as unpublished data from a pilot experiment, suggest that birds are the most common predators of *D. pumilio* at our study site (see also [14], [23], [24]); for this reason, and because our visual modeling is only applicable to avian predators, we focus the rest of this paper primarily on results for avian predation rates.

### Avian Visual Model Contrast-corrected Predation Rates

Most pairwise comparisons of replica color forms showed significant differences in contrast against the leaf litter background, regardless of the contrast method used (Fig. 2; Bonferroni corrected individual-test significance level α = 0.008; Table S1), underscoring the need to adjust expected attack rates for each replica color form based on differences in visual contrast. After contrast correction, we found that observed avian predation rates differed significantly from those expected based on replica color contrast alone (Fig. 3).

1. *Chromaticity contrast method (chromatic information only)*: Red replicas had higher contrast than yellow replicas; yellow replicas did not differ from brown replicas, but both yellow and brown replicas had greater contrast than black replicas (Fig. 2; Table S1). Avian attack rates differed from predictions based on visual contrast (Fig. 3; χ^2^_3_ = 21.49, *P*<0.001). Black and yellow replicas were attacked more often than expected (*black*: χ^2^_1_ = 8.54, *P = *0.004; *yellow*: χ^2^_1_ = 4.65, *P* = 0.031), brown replicas were attacked neither more nor less than expected (χ^2^_1_ = 0.21, *P* = 0.648), and red replicas were attacked less than expected (χ^2^_1_ = 8.08, *P* = 0.005). The attack rate for red replicas was significantly lower than that for yellow replicas after adjusting for differences in visual contrast (χ^2^_1_ = 13.79, *P*<0.001).
2. *Brightness contrast method (brightness information only)*: Yellow replicas had higher contrast than red replicas, which had higher contrast than brown, which had higher contrast than black (Fig. 2; Table S1). Avian attack rates differed from predictions based on visual contrast (Fig. 3; χ^2^_3_ = 94.83, *P*<0.001). Black and brown replicas were attacked at a higher rate than expected (*black*: χ^2^_1_ = 81.90, *P*<0.001; *brown*: χ^2^_1_ = 4.02, *P* = 0.045). Red replicas were attacked neither more nor less than expected (χ^2^_1_ = 0.28, *P* = 0.597). Yellow replicas were attacked less than expected (χ^2^_1_ = 8.64, *P* = 0.003). Red and yellow replicas did not differ in attack rates after adjusting for visual contrast (χ^2^_1_ = 1.85, *P* = 0.174).
3. *Composite contrast method (chromatic + brightness information)*: Yellow and red replicas had equal contrast, and both had higher contrast than brown, which had higher contrast than black (Fig. 2; Table S1). Avian attack rates differed from predictions based on visual contrast (Fig. 3; χ^2^_3_ = 23.39, *P*<0.001). Black replicas were attacked at a higher rate than expected (χ^2^_1_ **= **18.73, *P*<0.001). Brown and yellow replicas were attacked neither more nor less than expected (*brown*: χ^2^_1_ = 0.075, *P = *0.784; *yellow*: χ^2^_1_ = 0.205, *P* = 0.651). Red replicas were attacked less often than expected (χ^2^_1_ = 4.38, *P* = 0.036). Red and yellow replicas did not differ in their attack rates after adjusting for visual contrast (χ^2^_1_ = 1.80, *P* = 0.180).

## Discussion

From the perspective of the predator, a predation event consists of two components: (1) detection of prey, followed by (2) a behavioral decision to carry out an attack. Many experimental studies use attack rates on replicas to infer the second component - how do predators respond to different prey - without considering how differential detectability of prey types (i.e. the first component) may confound the inference. That is, do predators preferentially attack a prey type because they actually prefer that prey or merely because that prey type just happens to be more conspicuous? Because understanding predator behavior in the face of different prey types sheds light on the evolution of prey defense strategies like aposematism, polymorphism, and crypsis [33], teasing apart these two components of a predation event is crucial. To this end, laboratory studies are beginning to incorporate information from real visual systems to calculate how potential predators see their prey against a visual background [34], [35]. Our study is among the first to incorporate visual system data in a field predation study (but see [11]).

The absolute, uncorrected attack data from our study (Table 1) suggest that predators did not discriminate by prey type as each of the four replica morphs experienced similar predation pressure. However, this conclusion may be confounded by differences in prey detectability because the prey replica types did differ significantly from one another in conspicuousness to the avian eye against the forest floor background (Fig. 2). Indeed, after generating an expected attack distribution based on replica conspicuousness, we found that variation in attack rates did not match variation in conspicuousness (Fig. 3) – a deviation from expectation that suggests that avian predators are discriminating by prey type and that predators are making post-detection attack decisions that depend on the type of prey detected. This is an alternate conclusion from the one drawn from the uncorrected attack data, and it underscores the need to incorporate prey contrast into studies of predator response to novel prey.

Moreover, though attack rates often deviated from expectation (Fig. 3), we found that the direction of this deviation (i.e. whether predators attacked or avoided a prey type given conspicuousness) depended on which visual model we used to estimate prey contrast. For example, if predators use only chromatic information to detect their prey against a visual background, then the novel aposematic form was attacked at a higher rate than the familiar aposematic form (Fig. 3), which would be consistent with similar studies of avian predator behavior [7], [22], [36], [37] and suggest that La Selva’s avian predators will readily sample novel, conspicuous forms of *D. pumilio* if detected. However, if predators use brightness only or both brightness and chromaticity to detect prey, then the avian predator assemblage attacked the novel aposematic form at the same rate as the familiar aposematic form given conspicuousness, suggesting instead that La Selva avian predators either generalize their avoidance behavior learned from sampling the familiar aposematic form [10], [38] or possess an innate avoidance of small, brightly colored frogs [33]. We have no direct evidence favoring one model of avian vision over another (but see Text S2).

Thus, to answer whether avian predators at La Selva prefer or avoid novel prey, further research into avian visual thresholds is needed to determine what visual cues contribute to conspicuousness to the avian eye. Are birds using chromatic information only, brightness information only, or some additive (e.g. our study – composite method) or even non-linear (e.g. [38]) combination of the two to detect their prey? In conclusion, future studies should incorporate more sophisticated models of predator vision and contrast-perception to understand how predators behave in the face of novel prey.

## Supporting Information

Figure S1 **Reflectance values (mean ±1 s.d.).** *Top panel:* Red dorsum of the red-and-blue color morph of live *Dendrobates pumilio.* Spectrometer readings were taken from four live frogs and averaged to obtain one spectrum (data from T. Cronin, *pers. comm*). *Middle panel:* Red plasticine clay used in the local, aposematic replica. Three spectrometer readings were taken from each of five clay samples and were averaged to obtain one spectrum representing the red color. The clay and live spectra peak in roughly the same region, with the clay sample tailing off more quickly toward the end of the spectrum. The samples differ in brightness (note the y-axes), but achromatic differences are not generally a reliable method of discriminating colors under variable lighting conditions in the field; instead, chromatic differences are thought to be more important for discriminating colors in nature (Kelber et al. 2003). *Bottom panel*: Yellow plasticine clay used in the novel, aposematic replica. Three spectrometer readings were taken from each of five clay samples and were averaged to obtain one spectrum representing the yellow color.
(DOCX)

Figure S2 **Representative images of avian (A,B) and rodent (C) attacks.**
(DOCX)

Figure S3 **Leaf litter reflectance values (mean ±1 s.d.) from the thirteen transects.** There are thirteen transects because one trial was split into two locations. Each transect’s leaf spectrum was obtained by averaging spectra from four leaves collected from that transect. Three spectrometer readings were taken and averaged from each leaf.
(DOCX)

Figure S4 **Relative contrast estimates for the three visual contrast methods under a human visual model (scaled to 1) and human search time.** Estimates are not directly comparable among methods. The reciprocal of harmonic mean search time for each color is shown such that a large reciprocal value corresponds with a small mean search time (i.e. high contrast). Color forms assigned different letters differ significantly from each other after Bonferroni correction (Table S1). Colors follow the legend in Fig. 2.
(DOCX)

Table S1 **P-values for pairwise comparisons of visual contrast using the Wilcoxon signed-ranks test.** Both high and low values of V indicate significant differences between groups. The individual-test significance level after Bonferroni correction for six tests is α = 0.008.
(DOCX)

Text S1 **Human visual-model-based estimates of replica conspicuousness.**
(DOCX)

Text S2 **Evidence for chromatic cues being most important for visual contrast perception.**
(DOCX)
