# Supplementary figures and images for: Inferring Predator Behavior from Attack Rates on Prey-Replicas That Differ in Conspicuousness
Source: PLoS One. 2012 Oct 31;7(10):e48497. doi: 10.1371/journal.pone.0048497 (PMC3485355; doi:10.1371/journal.pone.0048497)

**FIGURE S1**

**
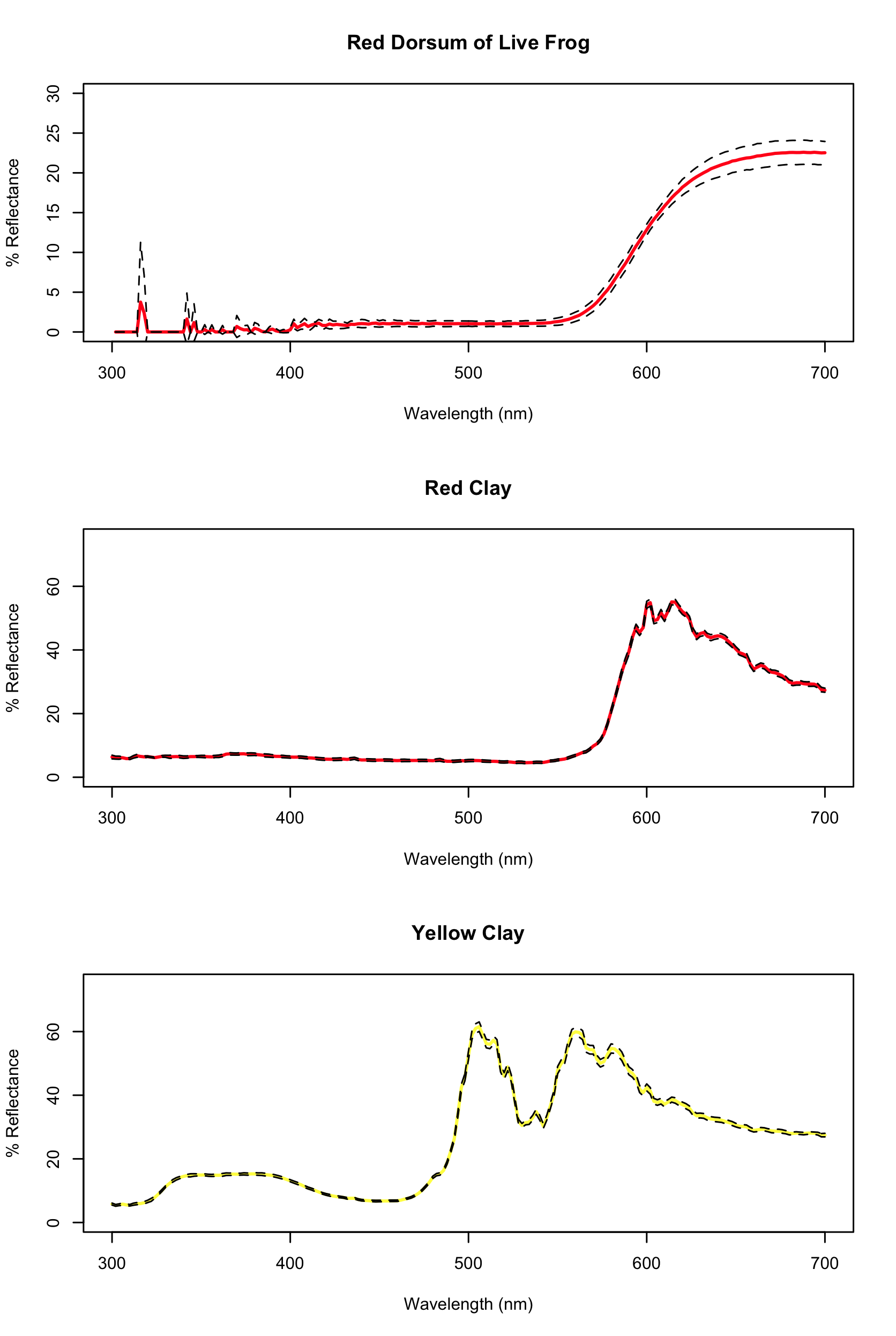
**

Supplement: Figure S1 — Reflectance values (mean ±1 s.d.). Top panel: Red dorsum of the red-and-blue color morph of live Dendrobates pumilio. Spectrometer readings were taken from four live frogs and averaged to obtain one spectrum (data from T. Cronin, pers. comm). Middle panel: Red plasticine clay used in the local, aposematic replica. Three spectrometer readings were taken from each of five clay samples and were averaged to obtain one spectrum representing the red color. The clay and live spectra peak in roughly the same region, with the clay sample tailing off more quickly toward the end of the spectrum. The samples differ in brightness (note the y-axes), but achromatic differences are not generally a reliable method of discriminating colors under variable lighting conditions in the field; instead, chromatic differences are thought to be more important for discriminating colors in nature (Kelber et al. 2003). Bottom panel: Yellow plasticine clay used in the novel, aposematic replica. Three spectrometer readings were taken from each of five clay samples and were averaged to obtain one spectrum representing the yellow color. (DOCX) [file pone.0048497.s001.docx]

**FIGURE S2**


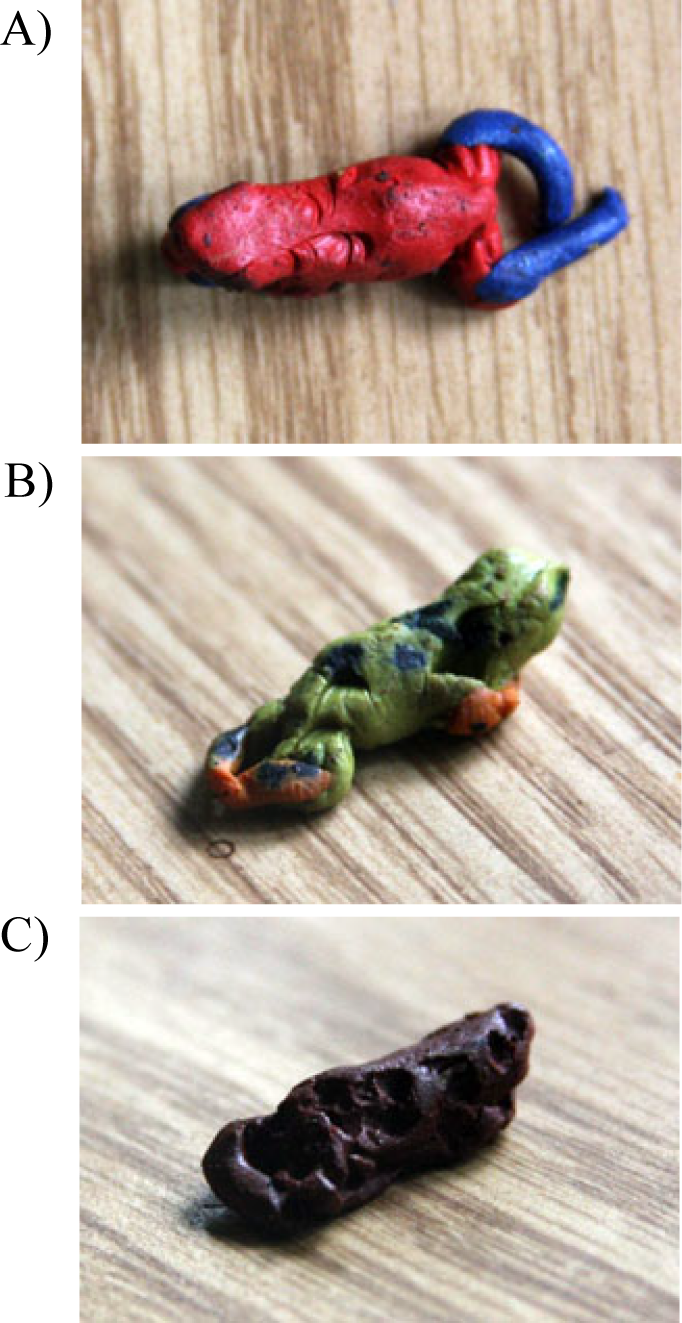

Supplement: Figure S2 — Representative images of avian (A,B) and rodent (C) attacks. (DOCX) [file pone.0048497.s002.docx]

**FIGURE S3**

**
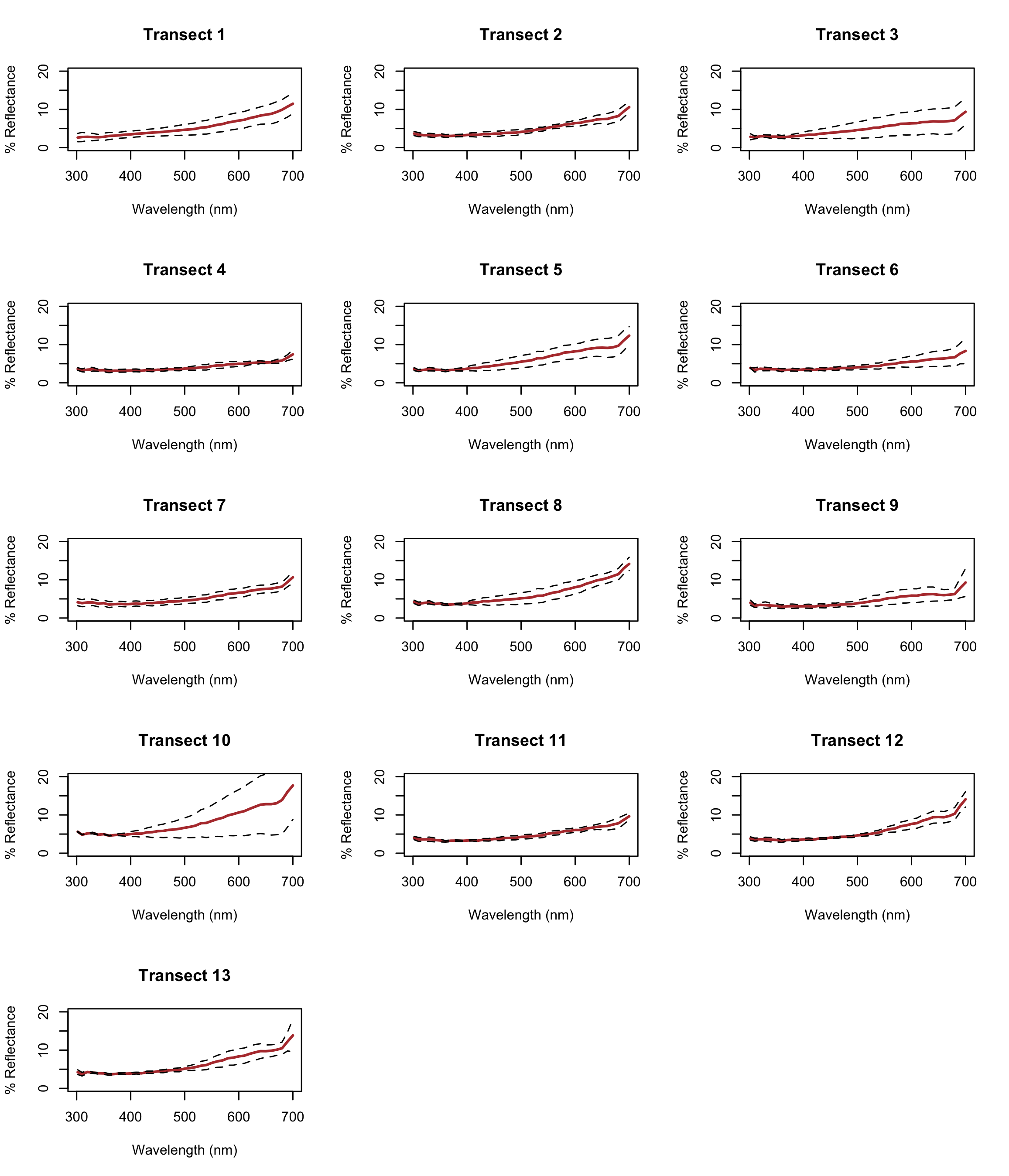
**

Supplement: Figure S3 — Leaf litter reflectance values (mean ±1 s.d.) from the thirteen transects. There are thirteen transects because one trial was split into two locations. Each transect’s leaf spectrum was obtained by averaging spectra from four leaves collected from that transect. Three spectrometer readings were taken and averaged from each leaf. (DOCX) [file pone.0048497.s003.docx]

**FIGURE S4**


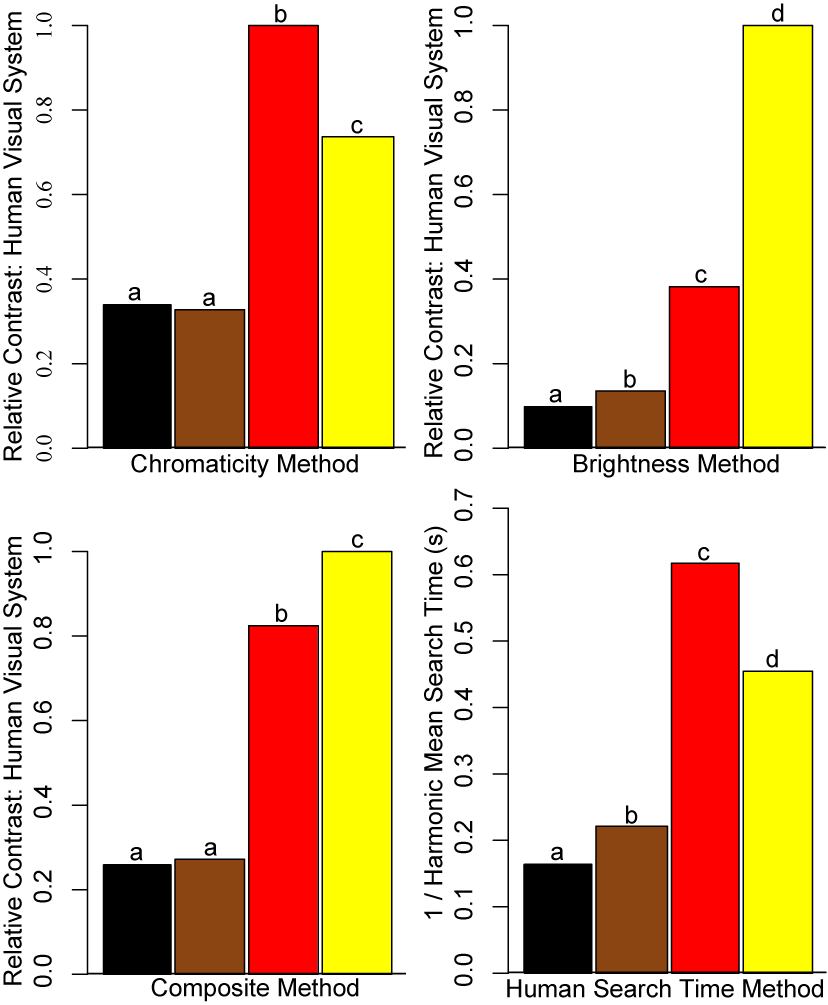

Supplement: Figure S4 — Relative contrast estimates for the three visual contrast methods under a human visual model (scaled to 1) and human search time. Estimates are not directly comparable among methods. The reciprocal of harmonic mean search time for each color is shown such that a large reciprocal value corresponds with a small mean search time (i.e. high contrast). Color forms assigned different letters differ significantly from each other after Bonferroni correction (Table S1). Colors follow the legend in Fig. 2. (DOCX) [file pone.0048497.s004.docx]
