# Supplementary material for: Inferring Predator Behavior from Attack Rates on Prey-Replicas That Differ in Conspicuousness
Source: PLoS One. 2012 Oct 31;7(10):e48497. doi: 10.1371/journal.pone.0048497 (PMC3485355; doi:10.1371/journal.pone.0048497)
Supplement: Table S1 — P-values for pairwise comparisons of visual contrast using the Wilcoxon signed-ranks test. Both high and low values of V indicate significant differences between groups. The individual-test significance level after Bonferroni correction for six tests is α = 0.008. (DOCX) [file pone.0048497.s005.docx]

**TABLE S1**

| visual model | contrast method | test | test statistic (V) | P-value |
| --- | --- | --- | --- | --- |
| avian | color | red vs. yellow | 1596 | <0.0001 |
| avian | color | red vs. brown | 1596 | <0.0001 |
| avian | color | red vs. black | 1596 | <0.0001 |
| avian | color | yellow vs. brown | 666 | 0.28 |
| avian | color | yellow vs. black | 1280 | 0.002 |
| avian | color | brown vs. black | 1214 | 0.0007 |
| avian | brightness | red vs. yellow | 0 | <0.0001 |
| avian | brightness | red vs. brown | 1595 | <0.0001 |
| avian | brightness | red vs. black | 1588 | <0.0001 |
| avian | brightness | yellow vs. brown | 1596 | <0.0001 |
| avian | brightness | yellow vs. black | 1596 | <0.0001 |
| avian | brightness | brown vs. black | 1367.5 | <0.0001 |
| avian | composite | red vs. yellow | 861 | 0.61 |
| avian | composite | red vs. brown | 1596 | <0.0001 |
| avian | composite | red vs. black | 1596 | <0.0001 |
| avian | composite | yellow vs. brown | 1596 | <0.0001 |
| avian | composite | yellow vs. black | 1595 | <0.0001 |
| avian | composite | brown vs. black | 1315 | <0.0001 |
| human | color | red vs. yellow | 1596 | <0.0001 |
| human | color | red vs. brown | 1596 | <0.0001 |
| human | color | red vs. black | 1596 | <0.0001 |
| human | color | yellow vs. brown | 1596 | <0.0001 |
| human | color | yellow vs. black | 1564 | <0.0001 |
| human | color | brown vs. black | 805 | 0.96 |
| human | brightness | red vs. yellow | 0 | <0.0001 |
| human | brightness | red vs. brown | 1542.5 | <0.0001 |
| human | brightness | red vs. black | 1536.5 | <0.0001 |
| human | brightness | yellow vs. brown | 1596 | <0.0001 |
| human | brightness | yellow vs. black | 1596 | <0.0001 |
| human | brightness | brown vs. black | 1360 | <0.0001 |
| human | composite | red vs. yellow | 2 | <0.0001 |
| human | composite | red vs. brown | 1596 | <0.0001 |
| human | composite | red vs. black | 1596 | <0.0001 |
| human | composite | yellow vs. brown | 1596 | <0.0001 |
| human | composite | yellow vs. black | 1596 | <0.0001 |
| human | composite | brown vs. black | 926 | 0.30 |
