# Supplementary material for: Inferring Predator Behavior from Attack Rates on Prey-Replicas That Differ in Conspicuousness
Source: PLoS One. 2012 Oct 31;7(10):e48497. doi: 10.1371/journal.pone.0048497 (PMC3485355; doi:10.1371/journal.pone.0048497)
Supplement: Text S1 — Human visual-model-based estimates of replica conspicuousness. (DOCX) [file pone.0048497.s006.docx]

**TEXT S1**

*Human Visual-model-based Estimates of Replica Conspicuousness*

*(i) A Field-based Proxy for Conspicuousness*

Because most replica studies of predator behavior rely on human assessments of color, we developed a field-based measure of replica conspicuousness to human observers – search time. While recovering the replicas at the end of each experimental trial, we recorded the number of seconds it took for a human researcher to locate each replica. The individual searching for frog replicas at the end of each experiment trial was not the same one who placed the replicas at the start of the experiment. If a replica was not found after 180 seconds, it was considered missing and was not included in subsequent analyses.

To test whether any of the three visual-model-based contrast estimates was a reasonable proxy for human search time, we adapted our avian visual-model-based approach to calculate visual contrast based on the human visual system. Human cone sensitivity functions (Stockman & Sharpe 2000) were used to calculate cone excitation values. The three contrast measures were calculated slightly differently for humans than for birds. First, because humans have only three cone types (birds have four), human-perceived colors can be plotted on a 2-dimensional triangular plane within Endler and Mielke’s (2005) tetrahedral color space. Second, humans do not have double-cones and seem to use input from all three cone types to evaluate brightness; therefore we summed the three cone excitation values to estimate brightness in the human visual system. The composite contrast measure was calculated identically in the human visual system as it was in the avian visual system. It is important to note that because of differences among visual systems, the absolute contrast values cannot be directly compared between human and avian visual systems, nor among the three contrast models (color, brightness, and composite).

*(ii) Analysis*

Search times could not be transformed to fit the distributional assumptions of ANOVA, so we used a Kruskal-Wallis test to ask whether search times differed among replica colors. We then used the Behrens-Fisher test, a nonparametric approach for assessing multiple pairwise comparisons (analogous to Tukey’s test; implemented in the R package “npmc”), to evaluate differences in human search time between replica colors.

We wished to test which estimate of contrast (color, brightness, or composite) in the human visual system provided the best fit to the human search time data – a field-based measure of replica contrast. Therefore we used the human contrast measures to parameterize three models (one for each contrast method) that predicted the search time for each replica color form while holding the total search time across the entire dataset constant. We calculated a likelihood score for each parameterized model to determine which measure of visual contrast best fit our search-time data.

We assumed that the distribution of search times would follow an exponential distribution, such that the instantaneous rate of replica detection would be constant for each replica color. The probability density function of the exponential distribution is:


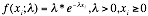
,

where λ is the instantaneous rate of detection for replica *i* and x*_i_* is the search time for replica *i*. The maximum likelihood estimate for
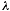
 is
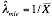
 where
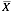
is the mean search time across all replica colors, regardless of replica contrast. However, we would like to find individual values of the instantaneous rate of replica detection,
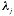
, for each replica color *j* (i.e.
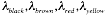
). With
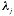
 for each replica color *j*, we can calculate the probability of observing a search time for a given replica according to an exponential distribution based on that replica’s color:


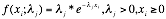
.

We wanted biologically reasonable values of λ*_j_* that maintain the difference in contrast among the replica colors. First, we calculated a constant
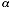
 as follows:


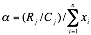
,

where
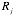
 is the number of recovered replicas of color *j*,
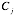
 is the contrast value for color *j*, *n* is the total number of recovered replicas, and
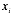
 is the search time for frog *i*. We then calculated
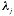
 as


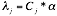
.

Calculating
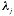
 in this manner preserves the relative contrast among replica colors because each contrast value is multiplied by a constant. Moreover, the mean of the expected values of the four exponential distributions parameterized by
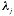
, *j* = {black, brown, red, yellow}, is equal to the expected value of the entire dataset parameterized by
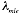
. Thus, the expected search time for each replica color is biologically reasonable and the expected values among colors represent the differences in contrast among replicas.

Once we have
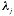
 for each color, we can calculate the probability of observing the data given the values of
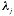
 using a standard log-likelihood framework:


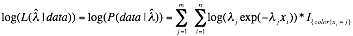
,

where
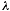
 is the vector (
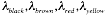
),
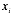
 is the search time for frog *i*, and
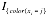
 is an indicator variable whose value equals 1 if the color of
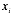
 is equal to *j*, and 0 otherwise. We assume that the
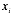
 are independently distributed. We calculated three separate
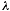
 and log-likelihood scores with replica contrast values from each of the three contrast models (color, brightness, and composite). Because each contrast model has the same number of parameters, the likelihood ratio test is not applicable and the Akaike Information Criterion model comparison will not add additional information. Thus, we are limited to comparing log-likelihood scores among the three models.

*(iii) Human Visual Model Contrast-corrected Predation Rates*

Recovery times for frog replicas were quite variable within colors. Because search times are expected to follow an exponential distribution (i.e. the probability of finding the frog per unit time is assumed to be constant), we report harmonic mean rather than arithmetic mean search times for each color form. Harmonic mean search time was 6.10s for black replicas, 4.52s for brown replicas, 2.20s for yellow replicas, and 1.62s for red replicas (Fig. S4). Mean search time differed significantly between each pair of color forms (Kruskal-Wallis Test: χ^2^_3_ = 735.66, *P* < 0.0001; post-hoc Behrens-Fisher test at α=0.05: all pairwise contrasts *P* < 0.001).

Nearly all pairwise comparisons of replica color forms differed significantly in their conspicuousness to the human visual system under each visual contrast method (Bonferroni corrected individual-test significance level α = 0.008; Fig. S4; Table S1). We calculated a likelihood score for the three contrast models that predicted search times for each replica. The color contrast model was the best fit to the observed human search-time data (log-likelihood = -7773.2), followed by the composite contrast model (log-likelihood = -7791.8) and the brightness contrast model (log-likelihood = -8259.6). There is no standard statistical method to test whether these models differ in their fit to the observed data because the models do not differ in the number of terms. A difference of 18.6 log-likelihood units between the best-fit (color) and next best-fit (composite) contrast models, however, corresponds to a 1.15x108-fold difference in likelihood.

REFERENCES

Endler JA, Mielke Jr. PW. 2005. Comparing entire colour patterns as birds see them. Biol. J. Linn. Soc. 86: 405-431.

Stockman A, Sharpe LT. 2000. The spectral sensitivities of the middle- and long-wavelength-sensitive cones derived from measurements in observers of known genotype. Vis. Res. 40: 1711-1737.
