# Supplementary material for: Inferring Predator Behavior from Attack Rates on Prey-Replicas That Differ in Conspicuousness
Source: PLoS One. 2012 Oct 31;7(10):e48497. doi: 10.1371/journal.pone.0048497 (PMC3485355; doi:10.1371/journal.pone.0048497)
Supplement: Text S2 — Evidence for chromatic cues being most important for visual contrast perception. (DOCX) [file pone.0048497.s007.docx]

**TEXT S2**

Several lines of evidence suggest that birds likely use color only when perceiving visual contrast. In learning trials with a food reward, Goldsmith et al. (1981) demonstrated that hummingbirds can be trained to use color cues to choose the correct feeder but cannot be trained to use brightness cues. Kelber et al. (2003) conclude that color, independent of brightness, is a more reliable signal for distinguishing objects against a background in conditions in which light intensity may vary depending on environmental conditions (see also Osorio and Vorobyev, 2005). Moreover, *D. pumilio* is widely regarded to be aposematic (Meyers and Daly, 1983; Saporito et al., 2007; Summers and Clough, 2001), so we predicted that La Selva predators would attack the familiar aposematic color replica less often than expected based on its conspicuousness because predators would already have experience with, and thus avoid, this form. The results from the color-only model are consistent with this expectation (Fig. 3 – note that the composite model is consistent with this prediction as well). Last, a separate analysis using the human visual system was best fit by the color-only model (see Text S1).

REFERENCES

Goldsmith TH, Collins JS, Perlman, DL (1981) A wavelength discrimination function for the hummingbird *Archilochus alexandri*. J Comp Phys A 143: 103-110.

Kelber A, Vorobyev M, Osorio D (2003) Animal colour vision – behavioural tests and physiological concepts. Biol Rev 78: 81-118.

Myers CW, Daly JW (1983) Dart-poison frogs. Sci Amer 248: 120-133.

Osorio D, Vorobyev M (2005) Photoreceptor spectral sensitivities in terrestrial animals: adaptations for luminance and colour vision. Proc Roy Soc Lond B Biol 272: 1745–1752.

Saporito RA, Zuercher R, Roberts M, Gerow KG, Donnelly MA (2007) Experimental evidence for aposematism in the dendrobatid poison frog *Oophaga pumilio.* Copeia 4: 1006-1011.

Summers K, Clough M (2001) The evolution of coloration and toxicity in the poison frogs. Proc Natl Acad Sci USA 98: 6227-6232
